# Marine dock pilings foster diverse, native cryptobenthic fish assemblages across bioregions

**DOI:** 10.1002/ece3.3288

**Published:** 2017-07-31

**Authors:** Simon J. Brandl, Jordan M. Casey, Nancy Knowlton, James Emmett Duffy

**Affiliations:** ^1^ Tennenbaum Marine Observatories Network Smithsonian Institution Edgewater MD USA; ^2^ Department of Invertebrate Zoology National Museum of Natural History Smithsonian Institution Washington DC USA

**Keywords:** biodiversity conservation, cryptobenthic fishes, dock pilings, endangered species, Latitudinal diversity gradient, local‐regional richness, marine urbanization

## Abstract

Anthropogenic habitats are increasingly prevalent in coastal marine environments. Previous research on sessile epifauna suggests that artificial habitats act as a refuge for nonindigenous species, which results in highly homogenous communities across locations. However, vertebrate assemblages that live in association with artificial habitats are poorly understood. Here, we quantify the biodiversity of small, cryptic (henceforth “cryptobenthic”) fishes from marine dock pilings across six locations over 35° of latitude from Maine to Panama. We also compare assemblages from dock pilings to natural habitats in the two southernmost locations (Panama and Belize). Our results suggest that the biodiversity patterns of cryptobenthic fishes from dock pilings follow a Latitudinal Diversity Gradient (LDG), with average local and regional diversity declining sharply with increasing latitude. Furthermore, a strong correlation between community composition and spatial distance suggests distinct regional assemblages of cryptobenthic fishes. Cryptobenthic fish assemblages from dock pilings in Belize and Panama were less diverse and had lower densities than nearby reef habitats. However, dock pilings harbored almost exclusively native species, including two species of conservation concern absent from nearby natural habitats. Our results suggest that, in contrast to sessile epifaunal assemblages on artificial substrates, artificial marine habitats can harbor diverse, regionally characteristic assemblages of vertebrates that follow macroecological patterns that are well documented for natural habitats. We therefore posit that, although dock pilings cannot function as a replacement for natural habitats, dock pilings may provide cost‐effective means to preserve native vertebrate biodiversity, and provide a habitat that can be relatively easily monitored to track the status and trends of fish biodiversity in highly urbanized coastal marine environments.

## 1 INTRODUCTION

Understanding the influences on the distribution of our planet's biodiversity has long formed a cornerstone of ecological investigation (Ricklefs & Schluter, 1993). With increasing human population size, anthropogenic biomes have emerged as a new opportunity to study and conserve biodiversity (McDonnell & Pickett, 1990; Niemelä, 1999). Initial assessments have revealed that even highly developed anthropogenic environments such as urban areas can harbor diverse communities, but that these communities often differ in their species composition compared to natural habitats (Blair, 1999; Chace & Walsh, 2006). Thus, it appears that the ever‐increasing landscape of anthropogenically influenced environments represents an opportunity and challenge for ecologists and conservationists alike (Gaston, Ávila‐Jiménez, & Edmondson, 2013; Grimm et al., 2008). Consequently, ecologists and conservation scientists have now embraced the opportunity to utilize terrestrial urban environments for large‐scale comparative research across multiple bioregions (Aronson et al., 2014) and the development of new management practices to aid the conservation of emerging biological communities and their ecosystem services (Dearborn & Kark, 2010). While this has resulted in improved understanding of biodiversity and ecology in urban landscapes (Aronson et al., 2014; Shochat et al., 2010), it is increasingly recognized that the cumulative nature of anthropogenic stressors in highly urbanized environments can lead to extremely homogenized, species‐poor assemblages that favor invasive species and opportunistic generalists (Blair, 1999; Grimm et al., 2008; McKinney, 2008).

Analogous to terrestrial developments, the disproportional presence of humans on the world's coastlines has resulted in “urbanized” coastal landscapes that are highly modified by human interference (Bulleri & Chapman, 2010; Dafforn et al., 2015). As a consequence, the response of marine biota to the replacement of natural structures with artificial, anthropogenic habitats (e.g., docks, breakwaters, jetties, and marinas) has garnered substantial attention, with many local‐scale studies demonstrating an overarching pattern similar to terrestrial systems: assemblages from artificial habitats are compositionally distinct (Bulleri & Chapman, 2010; Connell, 2001; Rogers, Byrnes, & Stachowicz, 2016) and frequently taxonomically homogenized (Airoldi, Turon, Perkol‐Finkel, & Rius, 2015; Bulleri & Chapman, 2010) in comparison with adjacent natural habitats. In sessile epifaunal assemblages, these patterns are often related to high proportions of invasive species (Bulleri, Chapman, & Underwood, 2005; Ruiz, Freestone, Fofonoff, & Simkanin, 2009), which appear to arise from differences in ecological processes such as predation, recruitment, competition, or herbivory between artificial and natural habitats (Ferrario, Iveša, Jaklin, Perkol‐Finkel, & Airoldi, 2016; Rodemann & Brandl, 2017; Rogers et al., 2016; Simkanin, Dower, Filip, Jamieson, & Therriault, 2013). However, in contrast to terrestrial urban ecology and with the exception of studies using submerged settlement tiles (e.g., Freestone & Inouye, 2015; Freestone, Osman, Ruiz, & Torchin, 2011; Freestone, Ruiz, & Torchin, 2013; Leray & Knowlton, 2015), relatively few assessments of biodiversity patterns in artificial, marine habitats such as docks have been performed that extend beyond local scales (but see Airoldi et al., 2015), which impedes our ability to soundly gauge the nature of biotic communities and their drivers in artificial marine habitats across geographic gradients.

Furthermore, the role of artificial habitats for the biodiversity of fishes is unclear. There is evidence that many mobile fishes are attracted to artificial structures (Rilov & Benayahu, 1998) and that these assemblages are compositionally distinct from nearby natural habitats (Clynick, Chapman, & Underwood, 2008). Yet, aside from differences in exposure and light regimes (Able, Grothues, & Kemp, 2013; Burt, Feary, Cavalcante, Bauman, & Usseglio, 2013; Cenci, Pizzolon, Chimento, & Mazzoldi, 2011), little is known about the reasons for these differences: intrinsic factors such as structure material, age, or the composition of the underlying epifauna appear to only have limited effects (Clynick, Chapman, & Underwood, 2007), despite the strong dependency of fishes on benthic communities for food and shelter (Clynick et al., 2007; Moreau et al., 2008). In addition, although invasive fish species demonstrably use artificial habitats (Jud, Layman, Lee, & Arrington, 2011; Lockett & Gomon, 2001), there is no broad evidence for increased proportions of nonindigenous fish species around artificial structures. Overall, our understanding of fish assemblages on artificial habitats may be compromised by the dominant use of underwater visual censuses (UVCs), which is the primary method to assess fish assemblages in many marine habitats (Cenci et al., 2011; Clynick et al., 2007, 2008; Rilov & Benayahu, 1998). The use of UVCs is known to significantly underestimate the abundance and diversity of cryptobenthic fishes (i.e., small, cryptic species with a tight association to the benthos [Ackerman & Bellwood, 2000; Clynick et al., 2007; Goatley & Brandl, 2017]), and UVCs are of limited use in low‐visibility environments.

The objective of the present study was to (1) provide the first description of cryptobenthic fish assemblages from artificial marine habitats across a large geographic scale, (2) to compare assemblages gathered from dock pilings and nearby natural habitats in Belize and Panama, and (3) to use the results to gauge the value of marine dock pilings for coastal vertebrate biodiversity across latitude. Specifically, using a SCUBA‐based sampling technique modified for dock pilings, we sampled cryptobenthic fish assemblages from 45 docks across six locations, covering 35 degrees of latitude in the Western Atlantic. We hypothesized that, if cryptobenthic fish assemblages consist of predominantly native species, assemblage‐level metrics (i.e., abundance, species richness, and diversity) of cryptobenthic fishes would be primarily determined by latitude and that assemblages across locations would be distinct. Regarding assemblages from dock pilings and nearby natural habitats in the tropics, we hypothesized that the community composition would vary between dock pilings and natural habitats.

## 2 MATERIALS AND METHODS

### 2.1 Study locations and sample size

**Figure 1:**
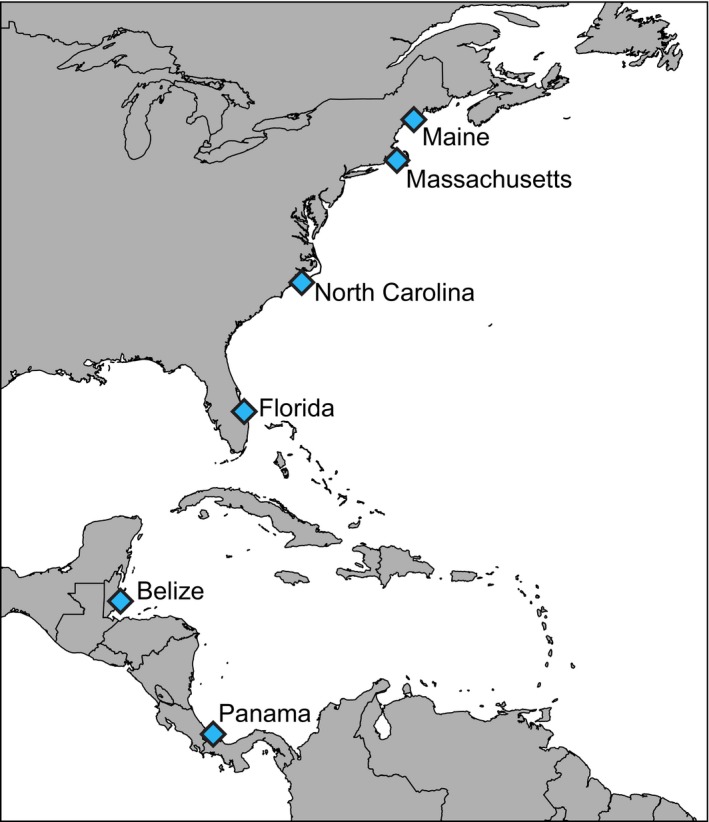
Map of the sampling locations utilized in this study. Eight docks were sampled in Bocas del Toro, Panama (9.35143°, −82.2571°), Carrie Bow Cay, Belize (16.8028°, −88.0834°), Fort Pierce, Florida (27.438889°, −80.335556°), and Beaufort, North Carolina (34.716667°, −76.650000°), seven docks in Falmouth, Massachusetts (41.551389°, −70.615278°), and six docks in Walpole, Maine (44.032778°, −69.518611°)

The study was conducted in six locations along the western Atlantic, covering 35 degrees of latitude from the Caribbean Sea to the Gulf of Maine (Fig. 1). Specifically, we sampled dock pilings in Panama, Belize, Florida, North Carolina, Massachusetts, and Maine for a total of 45 docks. For each dock, two separate pilings were sampled, which were pooled for all analyses. For each dock, we quantified the distance from the open ocean using free paths in Google Earth. Because other factors (broadly co‐varying with latitude) may influence cryptobenthic fish assemblages (e.g., population density, shipping traffic), we insured that docks within each location were spread across a variety of sites with varying abiotic factors. For example, docks in Panama ranged from Bocas town to the relatively pristine Punta Caracol (Easson, Matterson, Freeman, Archer, & Thacker, 2015), while docks in Belize were spread across inshore and outer‐shelf sites of the Belizean Barrier Reef, and docks in North Carolina ranged from the City pier of Beaufort to the Shackleford Banks (Fig. S1).

In addition, to compare assemblages from dock pilings with assemblages from natural substrata, we sampled 15 reef outcrops in Panama and Belize, which were spread throughout the Bay of Almirante, Panama, and the Belizean Barrier Reef, Belize (Fig. S2). Collections from natural habitats were restricted to the two tropical locations, as visibility, limited accessibility of habitats, and difficulty of sampling (in, for instance, mangroves in Florida) prevented the use of comparable techniques to sample cryptobenthic fishes in this study.

### 2.2 Field sampling

To sample cryptobenthic fishes from dock pilings, we modified an existing SCUBA‐based method that uses clove oil as an anesthetic (e.g., Ackerman & Bellwood, 2000; Ahmadia, Pezold, & Smith, 2012; Fig. S3). We used a fine mesh net (Delta Knotless Netting: 3 m × 3 m, 0.8 mm mesh size; Memphis Net & Twine Co.), fitted with two 120‐cm bungee cords attached on each end and a chain near the bottom of the net (approximately 30 cm above the bungee cord), as well as an impermeable tarp, also fitted with bungee cords. To carry out the sampling, we strung the net around the targeted piling using the bungee cords, insuring that there was enough slack to permit the formation of a trough in the net due to the weight of the chain. Next, we enveloped the net with the tarp and sprayed one liter of 5:1 ethanol clove oil solution (Jedwards International, Inc., Braintree, MA, USA) into the interior to anesthetize all fishes. Then, we removed the tarp, released the top bungee, and rolled the net from the top downward until reaching the bottom bungee cord. At this stage, we released the bottom bungee cord and rolled in the ends of the net to prevent the loss of organisms that fell into the trough. Subsequently, we brought the net to the surface and examined it for cryptobenthic fishes, which we immediately placed in an ice‐water slurry. Due to the elasticity of the bungee cords, the method can be applied to a variety of dock pilings independent of size, shape, or material of the pilings. In addition, the method can be applied reliably in low‐visibility environments (<10 cm visibility), which are often typical for inshore ecosystems and prevent traditionally employed methods such as UVCs.

To standardize fish assemblages by the size of the piling, we estimated the surface area (SA; SA = 2π*rh*) of the sampled pilings (mean surface area: 2.33 ± 0.13 m^2^ *SE*). To do so, we measured piling circumference and length and calculated the piling's radius from its circumference (*C* = 2π*r*). In addition, we obtained samples of the sessile benthic community on each dock using a 20 × 20 cm quadrat and a paint scraper. Each sample was dried at 60°C in a drying oven for at least 24 hr and weighed to the nearest milligram. Biomass was averaged across the four samples for the analyses.

To sample reef outcrops, we used enclosed clove oil stations (following [Ackerman & Bellwood, 2000; Ahmadia et al., 2012; Goatley, González‐Cabello, & Bellwood, 2016]). To estimate the surface area of reef outcrops (mean surface area: 4.42 ± 0.25 m^2^ *SE*), we measured curved surface length (CSL), derived the outcrop's radius (*r* = 2CSL/2π), and subsequently calculated available surface area assuming reef outcrops represent idealized hemispheres (SA = 4π*r* ^2^/2).

In the laboratory, we photographed all fishes laterally in a small photo‐tank using a Nikon D300 DSLR camera fitted with an AF‐S Micro Nikkor 60 mm lens (f/2.8G ED; Nikon Inc., Melville, NY, USA). We then identified all fishes to the level of species or morphotypes within genera (using [Robertson & Van Tassell, 2015]). Finally, we measured all fishes to the nearest 0.1 mm (SL and TL) using digital calipers and placed them in 95% ethanol for preservation.

### 2.3 Analyses

First, we calculated cryptobenthic fish density for each dock by dividing abundance by the log of the sampled surface area in order to investigate the overall number of individuals pilings support in various locations. Then, we used fixed‐coverage‐based subsampling (Chao & Jost, 2012; Hsieh, Ma, & Chao, 2016) to extrapolate or rarefy species richness estimates at each dock to a sample completeness of 90% (i.e., a less than ten percent chance that a newly detected individual will represent a species not sampled previously). Docks for which this threshold resulted in unreliable extrapolations (due to an insufficient number of species) were excluded from the analysis (one dock in Belize, North Carolina, and Maine; two docks in Massachusetts). Using the fixed‐coverage estimates of species richness, we calculated species density by dividing the estimates by the log of sampled surface area. To detect potential trends in the importance of rare or highly abundant species across latitude, we repeated the rarefaction procedure to obtain coverage‐based estimates of diversity using Simpson's D. All subsequent analyses were performed on the fixed‐coverage estimates.

To determine the effect of *Latitude* on fish density, species density, and Simpson diversity, we used three linear mixed models with each dock as a replicate, with a random intercept specified for *Location* as a grouping factor. All three response variables were log‐transformed prior to the analysis because they consisted of strictly positive, continuous data that do not conform to the assumptions of the normal distribution. We included distance from the open ocean (*Distance*) and the average weight of the sessile community scraped from the pilings (*Benthos*) as fixed effects given their potential influence on cryptobenthic fish assemblages. All fixed effects were scaled and centered to facilitate comparison of effect sizes. For the density model, a second‐order polynomial was specified. For all models, homoscedasticity of variance was verified by plotting the residuals against the fitted values (Fig. S4).

To compare cryptobenthic fish assemblages between natural and artificial habitats in Panama and Belize, we performed three linear models, again using the logarithm of total fish density and the log‐transformed fixed‐coverage estimates of species density and Simpson's D as the response variables. We specified location (Panama or Belize) and habitat type (natural vs. artificial) as well as their interaction as fixed effects. For all models, we assessed homogeneity of variances by plotting the fitted model values against the model residuals (Fig. S5). We examined model fits visually by calculating predicted model values and superimposing the predicted mean (±95% confidence intervals) on the raw values.

### 2.4 Community composition

In order to determine whether cryptobenthic fish assemblages are regionally characteristic or largely homogenized across locations, we compared the community composition of docks across locations using a classical metric multidimensional scaling ordination (cMDS) on Bray‐Curtis dissimilarities across docks. We then tested for spatial correlations in the community composition using a Mantel test. To do so, we calculated a matrix of geographic distance among all docks and regressed the geographic distance matrix against a Bray‐Curtis dissimilarity matrix of docks based on their cryptobenthic fish communities. As the conformance of data to a bivariate normal distribution was not confirmed, we used the rank‐based Spearman statistic (ρ) to determine spatial correlation.

To visualize community composition across habitats and locations in Belize and Panama, we performed a nonmetric multidimensional scaling ordination (nMDS) using Bray‐Curtis distances of the square‐root‐transformed species matrix. To formally compare communities, we used a second PERMANOVA and SIMPER analysis. Finally, to compare cryptobenthic fish communities from docks constructed with different materials in Belize and Panama (all docks in the United States were exclusively wooden, whereas the docks in Panama and Belize were constructed with either wood, PVC, cement, or iron), we performed a third PERMANOVA with square‐root‐transformed Bray‐Curtis distances using location and the dock piling material as fixed effects. As only one iron dock piling was sampled during the study, we excluded this dock from the material analysis. We visualized the results using an nMDS ordination. All analyses were performed using the software *R* and the packages *vegan* (Oksanen et al., 2007), *lme4* (Bates, Maechler, Bolker, & Walker, 2015), and *iNEXT* (Hsieh et al., 2016).

## 3 RESULTS

Overall, we sampled 1,303 individuals, representing at least 59 species, of cryptobenthic fishes from marine dock pilings from Panama to Maine, and we sampled 2,528 individuals, representing at least 106 species, from reef outcrops in Belize and Panama. For dock pilings, regional (γ) diversity was highest in Belize (21 species), followed by Florida (19 species), Panama (14 species), North Carolina (10 species), and Massachusetts and Maine (both with four species).

### 3.1 Density, species density, and diversity

**Figure 2:**
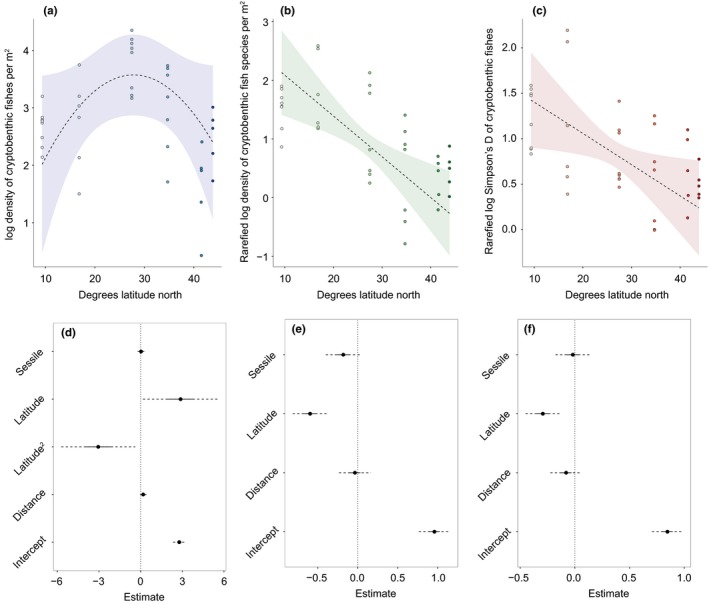
The effect of latitude, distance to open ocean, and sessile biomass on cryptobenthic fish density, species density, and Simpson's D. (a)–(c) represent scatterplots of raw values plotted against latitude with model fits obtained from linear mixed models (± their 95% confidence intervals) superimposed. (d)–(f) represent the normalized associated model parameter estimates (± their 95% confidence intervals) for all parameters. Different shades represent different locations ranging from Panama (lightest) to Maine (darkest)

The density of cryptobenthic fishes exhibited a unimodal distribution across latitude with a peak at approximately 28° latitude north, which corresponds to our sampling location at Fort Pierce, Florida (Fig. 2a,d). In the linear mixed model (marg. *R* ^*2*^ **=* 0*.29), the polynomial fitted for *Latitude* was the only significant factor (*t *=* *−2.24, *df *=* *29, *p *=* *.03) with a parameter estimate (β) of −3.05 (±1.36 *SE*). None of the other parameters were significant, although the effect of the linear parameter for *Latitude* was only marginally weaker than the polynomial (β = 2.89 ± 1.38 *SE*;* t *=* *2.08, *df *=* *29, *p *=* *.05). In contrast, species density of cryptobenthic fishes exhibited a clear, linear negative relationship with *Latitude* (β = −.59 ± .11 *SE*;* t *=* *−5.37, *df *=* *30, *p *<* *.0001), while both *Distance* and *Benthos* had no effects of species density (Fig. 2b,e). The marginal *R* ^*2*^ for the species density model was 0.44. The model for Simpson's D was generally similar to species density, showing a clear, linear negative relationship with *Latitude* (β = −.29 ± .08 *SE*;* t *=* *−3.64, *df *=* *30, *p *=* *.001) and no effect of *Distance* or *Benthos* (Fig. 2c,f). However, the explanatory power of the model was lower for Simpson's D compared to species density (marginal *R* ^*2*^ **=* *0.30). Using raw values of species density and Simpson's D instead of fixed‐coverage subsamples weakened the relationships between the respective indices and latitude, but did not change the results significantly (Fig. S6). Homogeneity of variance was confirmed for all models and predicted fits showed adequate model performance when superimposed on the raw data (Fig. 2).

**Figure 3:**
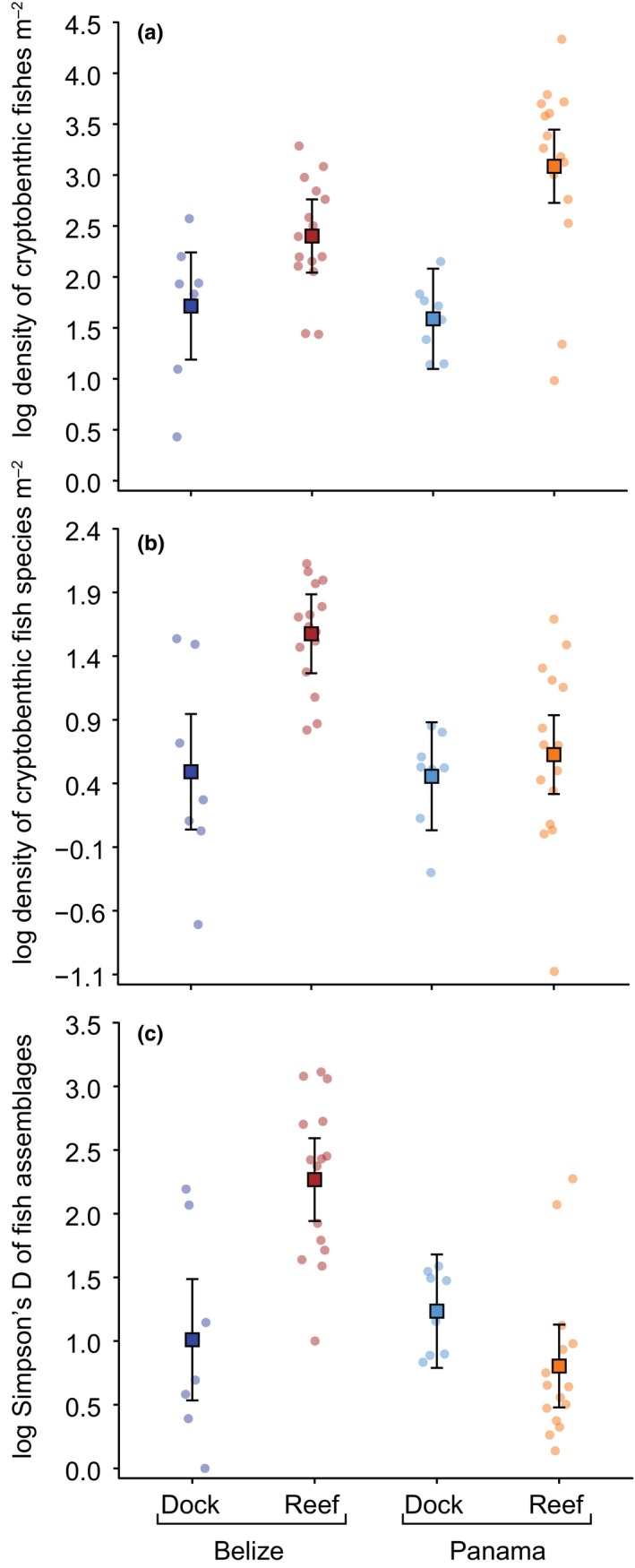
Density, rarefied species density, and rarefied Simpson's D of cryptobenthic fishes on docks and coral reef outcrops in Belize and Panama. Whisker plots represent the mean predicted model value for each group (± their 95% confidence intervals), while superimposed dots represent raw values. A horizontal random jitter was applied to the raw data to facilitate interpretation

When comparing the assemblages collected from dock pilings and reef outcrops, the models revealed significant differences for total fish density, species density, and Simpson's D (Fig. 3). We found significantly fewer fishes and fewer species per square meter on dock pilings compared to reef outcrops; however, only reef outcrops in Belize had significantly more species than dock pilings in either location, while reef outcrops in Panama were statistically indistinguishable from dock pilings. Similarly, reefs in Belize were highest in terms of Simpson's D, but there were no significant differences between reef outcrops in Panama and dock pilings in Belize and Panama (Table 1).

**Table 1:** Model parameter estimates and their 95% confidence intervals for the models comparing density, coverage‐based species density, and Simpson's D among docks and reef outcrops in Belize and Panama

	Density (individuals per m^2^)			Species density (species per m^2^)			Simpson's D (unitless)		
Parameter	Est.	LCI	UCI	Est.	LCI	UCI	Est.	LCI	UCI
Docks‐Belize (ref)	1.71	1.19	2.24	0.49	0.04	0.95	1.01	0.53	1.49
Panama	*−0.12*	*−0.84*	*0.59*	*−0.40*	*−0.66*	*0.58*	*0.22*	*−0.42*	*0.88*
Outcrops	**0.68**	**0.05**	**1.32**	**1.08**	**0.53**	**1.63**	**1.25**	**0.68**	**1.83**
Outcrops‐Panama	0.81	*−*0.07	1.69	*−* **0.91**	*−* **1.67**	*−* **0.15**	*−* **1.69**	*−* **2.49**	*−* **0.89**
Adjusted *R* ^*2*^	**0.40**			**0.38**			**0.49**		

For each model, dock pilings in Belize represent the reference against which parameter estimates are provided. Significant parameters (for which 95% CIs do not intersect 0) are in bold. Analyses were conducted on scaled and centered data so the coefficients are standardized.

LCI, lower confidence interval; UCI, upper confidence interval. Italicized values should be in normal font.

### 3.2 Community composition

**Figure 4:**
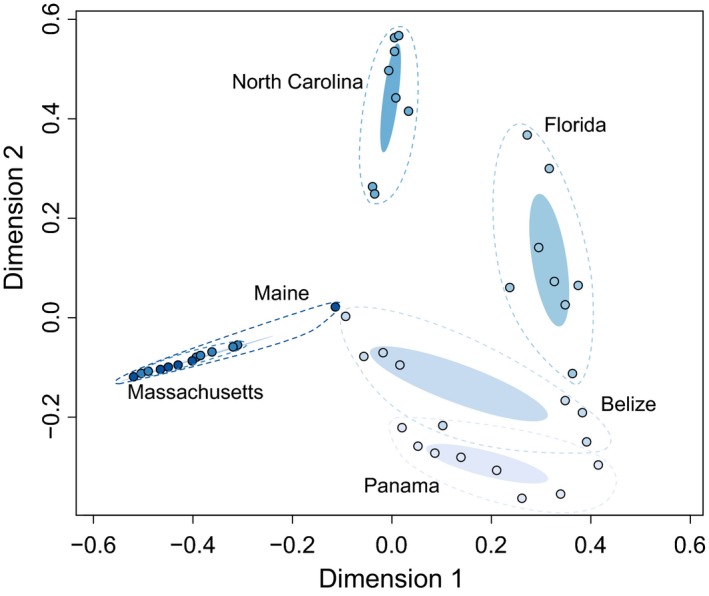
Classical metric multidimensional scaling ordination of cryptobenthic fish assemblages from dock pilings across six locations. Solid ellipses represent the standard deviation of the weighted mean‐averages for each location and their 95% confidence intervals, while dashed ellipses encapsulate all samples of a given location. Shadings correspond to Figure 2a, ranging from Panama (lightest) to Maine (darkest). Except for Maine and Massachusetts, for which cryptobenthic fish assemblages broadly overlap, all locations exhibit distinct, regionally characteristic species assemblages

There was a significant correlation between geographic distance and cryptobenthic fish community composition (Mantel test statistic: *r *=* *.64, *p *<* *.001). This was further supported by the ordination yielded by the cMDS, which revealed clear separation of communities across locations, with the exception of communities from Massachusetts and Maine (Fig. 4). The species present in each location are provided in Table S1.

**Figure 5:**
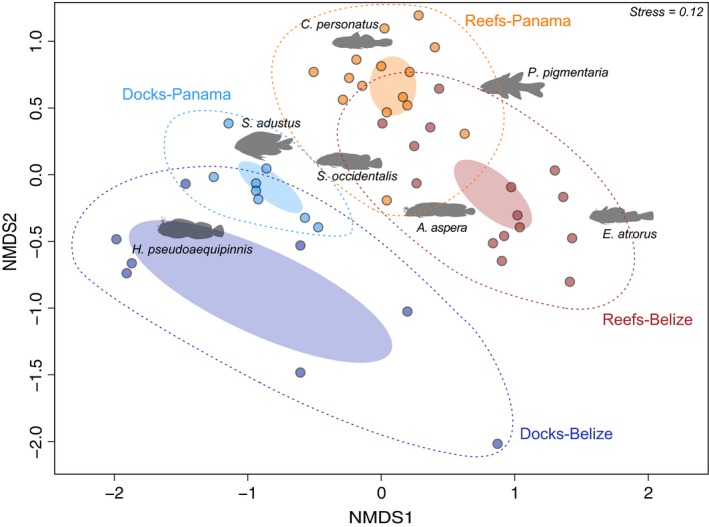
nMDS ordination of cryptobenthic fish communities on docks and reef outcrops in Belize and Panama. Assemblages are distinct (ANOSIM: pseudo‐*p* < .001), suggesting that communities on dock pilings are no more homogenous than on natural substrata. Solid ellipses represent the standard deviation of the weighted mean‐averages for each group and their 95% confidence intervals, while dashed ellipses encapsulate all samples of a given group. The seven most influential species are superimposed in black letters. Light blue* *=* *Docks‐Panama; dark blue* *=* *Docks‐Belize; Orange* *=* *Reefs‐Panama; Red* *=* *Reefs‐Belize

The habitat PERMANOVA showed that communities from dock pilings and reefs in Panama and Belize were distinct in species composition (Location*Habitat:* F *=* *5.73, *p *<* *.001), although differences among habitats were strongest (*Habita*t: *F *=* *12.62, *p *=* *.001; *Location*:* F *=* *7.39, *p *=* *.001). This was supported by the nMDS ordination, which showed distinct ellipses for all four communities, with a greater distance between the two habitat types than the two locations (Fig. 5). Across all comparisons, seven species of cryptobenthic fishes represented the most influential species (Table S2). The material PERMANOVA revealed no significant effect of construction material on community composition (Material: *F *=* *1.42, *p *=* *.15) while location had a significant effect (Location: *F *=* *3.63, *p *=* *.001). This was also supported by the nMDS ordination (Fig. S7). However, given the relatively low sample size for each material (cement: *n* = 5; wood: *n* = 6; PVC: *n* = 4), the potential effect of material on fish communities requires further testing.

**Figure 6:**
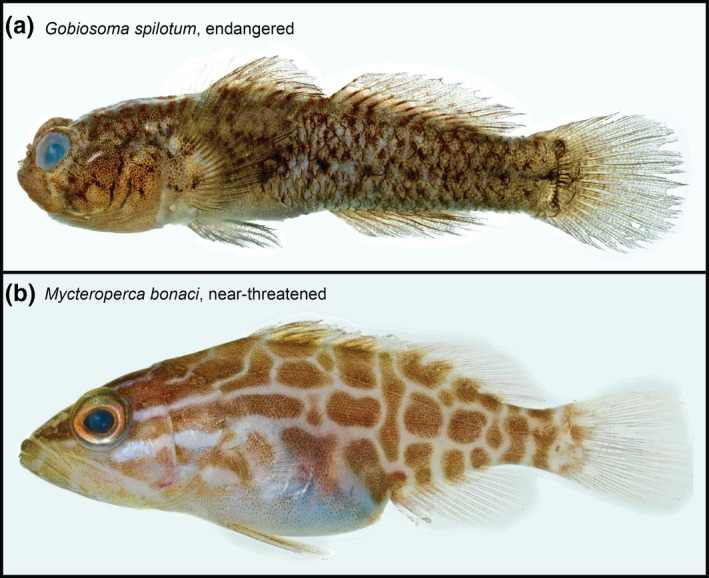
The goby *Gobiosoma spilotum*, sampled from a dock in Bocas Town, Bocas del Toro, Panama, and the serranid *Mycteroperca bonaci*, sampled from a dock on Southwater Caye, Belize. The conservation status of these species is categorized as endangered and near‐threatened, respectively, suggesting that dock pilings may provide valuable habitat for native vertebrate biodiversity in heavily urbanized areas

Finally, among all individuals sampled from dock pilings across latitudes, only one individual of the invasive lionfish *Pterois volitans* was caught on a dock in Fort Pierce, while four individuals of *P. volitans* were collected from reef outcrops in Belize and Panama. In contrast, we collected one individual of *Gobiosoma spilotum*, an endangered species previously known only from the Panama Canal area, on a dock in Bocas del Toro (well outside its known range). In addition, we also sampled two individuals of the near‐threatened species *Mycteroperca bonaci* on docks in Belize and Florida (Fig. 6).

## 4 DISCUSSION

Here, we provide the first comparative, large‐scale study of marine vertebrate communities from artificial habitats. We demonstrate that the diversity of cryptobenthic fish assemblages on marine dock pilings decreases with increasing latitude (albeit limited to six distinct locations), following the Latitudinal Diversity Gradient (LDG, Pianka, 1966; Hillebrand, 2004) and corroborating the only other large‐scale biogeographic study on cryptobenthic fishes, which showed conformance to preconceived gradients in diversity between the Indo‐Australian Archipelago and the Eastern Pacific (González‐Cabello & Bellwood, 2009). We further show that, although less diverse and densely populated than nearby coral reef outcrop habitats in tropical locations, marine dock pilings harbor diverse, abundant, regionally characteristic assemblages of native cryptobenthic fish species, including species of conservation concern. Based on these findings, we propose that marine dock pilings provide a readily utilized habitat that may contribute to the preservation of local vertebrate biodiversity in highly urbanized areas.

### 4.1 Biodiversity of cryptobenthic fishes from marine dock pilings

Although insufficient knowledge about distribution patterns of cryptic biotic assemblages impedes assessments of how regional or local factors influence observed diversity patterns (Leray & Knowlton, 2015), our findings permit inferences about the processes shaping community assembly on dock pilings (Srivastava 1999). For example, there was a slight divergence between regional and local‐scale estimates of species richness. Specifically, dock pilings in Panama (*n* = 14) exhibited substantially lower regional species richness than Belize (*n* = 21) and Florida (*n* = 19), while harboring a local (i.e., dock‐specific) species richness of 5.56 species (mean ± 0.77 *SE*), which is equal to or higher than average local estimates for Belize (mean* *=* *5.71 ± 1.46 *SE*) and Florida (mean* *=* *4.86 ± 1.20 *SE*), respectively. This suggests that assemblages in Florida and Belize may be saturated, corresponding to a type II model of local‐regional richness relationships (i.e., ecological constraints prevent colonization of pilings by all species regionally present, suggesting strong local effects) rather than a type I model (i.e., the majority of species from the regional pool are present on each dock, suggesting strong regional effects, Ricklefs & Schluter, 1993; Gaston, 2000). This is further supported by the substantial variability in species richness among docks at most locations (Fig. 3), a pattern previously cited in support for the significance of local processes (Osman, 2015). However, while our results provide important baseline information pointing toward the importance of local factors for community assembly, further work will be needed to establish independent regional inventories of cryptobenthic fishes from dock pilings to disentangle the processes that shape biotic communities in these emergent habitats.

### 4.2 Macroecological patterns

Given the strong effect of latitude and the spread of docks across sites with varying levels of urbanization in each location, it appears that latitude (and its environmental, geohistorical, or geometric covariates) is the most parsimonious driver of cryptobenthic fish community structure on dock pilings, despite the limited number of locations sampled. Thus, our findings suggest that the diversity of cryptobenthic fishes from dock pilings follows well‐established biogeographic patterns found in natural habitats such as the Latitudinal Diversity Gradient (Hillebrand, 2004; Pianka, 1966), which may indicate that assemblages are subject to relatively “natural” ecological processes (Leray & Knowlton, 2015). For example, natural communities of prosobranch gastropods in the Western Atlantic exhibit the steepest decline in diversity between approximately 20° and 40° latitude (Roy, Jablonski, Valentine, & Rosenberg, 1998), while diversity in locations beyond these latitudes is relatively even across the next 10°–20°. Although limited to six locations, fish communities from dock pilings appear to corroborate this pattern, with the sharpest decline occurring from Belize (ca. 17°) to Massachusetts (ca. 41°), while Panama and Belize and Massachusetts and Maine were broadly comparable. This demonstrates a likely contrast between sessile epifauna and cryptobenthic fish communities on artificial substrata, as previous studies using settlement panels and their associated fouling communities found no gradient in biodiversity from Panama to Connecticut (Freestone et al., 2011).

Compared to diversity patterns, relatively little is known about the determinants of biomass and productivity in coastal marine habitats across latitude. The peak of fish density at mid‐latitudes (Florida and North Carolina) in the present study may result from the characteristics of the two systems (the Indian River Lagoon, IRL and the Beaufort Inlet, BI) instead of latitudinal patterns. In both locations, the assemblages were numerically dominated by three blenniid species, which in combination represented 83.6% (*Hypleurochilus geminatus*,* H. pseudoaequipinnis*, and *Scartella cristata* in Florida) and 94.8% (*H. geminatus* in North Carolina) of the obtained specimens. Notably, all three species are largely herbivorous (Hundt et al. 2017 Robertson & Van Tassell, 2015), which suggests that their abundance depends strongly on system‐wide productivity and the availability of benthic algae on the pilings. Both the IRL and the BI are large coastal lagoons that rank as some of the most productive marine habitats worldwide (Kennish & Paerl, 2010); thus, they potentially provide abundant primary resources to sustain large populations of the three blenniid species. In contrast, although *H. pseudoaequipinnis* was present in both Belize and Panama, the pilings there may be less suitable for this species to achieve high abundance. The Belizean Barrier Reef is a tropical, oligotrophic system with relatively little primary productivity (Rützler & Macintyre 1982), which potentially limits population size in this location. Conversely, although the Bay of Almirante in Panama is also a productive coastal lagoon (D'Croz, Del Rosario, & Gondola, 2005), pilings in this location were heavily overgrown by sponges instead of algae; in contrast to algae, sponges are not a common food resource for cryptobenthic fishes, possibly contributing to lower densities of herbivorous fishes in Panama (Robertson & Van Tassell, 2015). Future studies that incorporate additional location‐ and dock‐specific biotic and abiotic factors may shed light on local or regional determinants of fish density that may also help explain the large dock‐specific variability within locations.

### 4.3 Artificial versus natural habitats

Although the biotic composition of artificial substrata has been studied extensively in the last two decades, little consensus has been achieved on overarching patterns due to a lack of large‐scale, synthetic approaches that go beyond the deployment of experimental settlement plates (Freestone & Inouye, 2015; Freestone et al., 2011, 2013). In cryptobenthic fish assemblages, we found that in both Belize and Panama, population density and species density were significantly lower on dock pilings than on surrounding reef outcrops, and that the community composition differed strongly between dock pilings and reef outcrops (cf. Bulleri & Chapman, 2010; Clynick et al., 2008; Connell, 2001). However, the cause for dissimilarity between artificial and natural habitats appears to vary distinctly among broad taxonomic groups. For sessile epifauna, the most frequently cited reason is the high prevalence of invasive species on artificial substrata, which is linked to differences in biotic interactions between natural and artificial habitats (Rodemann & Brandl, 2017; Rogers et al., 2016; Ruiz et al., 2009; Simkanin et al., 2013). Furthermore, orientation, slope, and material of the artificial structure are also cited as important agents for homogenization of invertebrate assemblages (Glasby & Connell, 2001), with the availability of microtopographic refuges being particularly important for sessile organisms (Brandl & Bellwood, 2016; Freestone & Osman, 2011). In contrast, compositional differences among mobile fish assemblages appear to be largely due to different macro‐habitats (sheltered vs. exposed [Clynick et al., 2008; Burt et al., 2013; ]), light availability (Able et al., 2013), or ontogenetic shifts in habitat preferences (Fowler & Booth, 2013). For the cryptobenthic fish assemblages examined in this study, no invasive species were sampled from docks, and the material with which docks were constructed had a limited effect on community composition. Similarly, both docks and reefs contained a considerable proportion of juvenile and subadult individuals of both mobile and cryptobenthic fishes. Therefore, compositional differences in cryptobenthic fish assemblages between dock pilings and reef outcrops may be due to depth, the surrounding habitat, or the strictly vertical orientation of dock pilings (Clynick et al., 2008; Rilov & Benayahu, 1998). A formal comparison of communities from artificial and natural substrates beyond the tropics may aid our understanding.

### 4.4 Gauging the value of dock pilings for vertebrate biodiversity

The value of artificial structures for coastal biodiversity has frequently been questioned based on the presence of nonindigenous species and the apparent capacity of nonindigenous species to spread along corridors provided by artificial structures (Airoldi et al., 2015; Glasby & Connell, 2001). In contrast, our findings indicate that dock pilings may provide valuable habitat for cryptobenthic fishes in urbanized coastal environments. Despite high potential for biological invasions by cryptobenthic fishes due to their small size, crypsis, and habitat requirements (Wonham, Carlton, Ruiz, & Smith, 2000), we found almost exclusively native, regionally characteristic species of cryptobenthic fishes on dock pilings across the northwestern Atlantic (with the lionfish *Pterois volitans* as the only detected nonindigenous species). In fact, the detection of two species currently listed as near‐threatened and endangered under the IUCN Red List suggests that dock pilings may constitute a habitat type that, beyond fostering local biodiversity, also harbors rare species of conservation concern and contributes to the maintenance of their declining populations (Fig. 6; Van Tassell, Aiken, & Tornabene, 2015). Comparable findings have been reported for terrestrial, urban environments (McKinney, 2008), highlighting that habitat provision by anthropogenic structures can benefit species conservation in areas where undisturbed natural habitat is scarce. As the provision of habitat is a mere by‐product of dock pilings (which are commonly constructed to serve human interests), our results suggest that the cost of dock pilings in the context of biodiversity conservation is minimal. Based on our findings and previous research (Clynick et al., 2007), it appears that the material of pilings is of secondary importance for fish communities, suggesting that the most permanent solutions (such as PVC pilings) may be preferable, as they will prevent frequent replacement or maintenance of pilings.

Finally, from an ecological perspective, the ubiquity and prevalence of dock pilings in coastal areas and their ease of access combined with the demonstrated presence of native, regionally characteristic fish communities may permit the efficient monitoring of heavily developed, urbanized environments. Specifically, although dock pilings can certainly differ considerably in their inherent characteristics (such as age, material, or size), they represent a relatively standardized habitat that consists of confined, vertical surfaces and are available in coastal regions worldwide. Thus, extensive sampling of cryptobenthic fishes from marine dock pilings may provide insights into biodiversity trends and patterns of trophically important species (Depczynski & Bellwood, 2003) in areas that are often missing from large‐scale datasets (Dornelas et al., 2014). This is particularly interesting because cryptobenthic fishes are known to be highly sensitive to environmental changes (Ahmadia et al., 2012; Goatley et al., 2016), which may be the cause for the extensive dock‐specific variation found in the present study.

## CONFLICT OF INTEREST

None declared.

## AUTHOR CONTRIBUTIONS

SJB, NK, and JED developed the study; SJB and JMC collected the data; SJB analyzed the data; SJB wrote the first draft of the manuscript and all authors contributed thereafter.

## References

[ece33288-bib-0001] Able, K. W. , Grothues, T. M. , & Kemp, I. M. (2013). Fine‐scale distribution of pelagic fishes relative to a large urban pier. Marine Ecology Progress Series, 476, 185–198.

[ece33288-bib-0002] Ackerman, J. L. , & Bellwood, D. R. (2000). Reef fish assemblages: A re‐evaluation using enclosed rotenone stations. Marine Ecology Progress Series, 206, 227–237.

[ece33288-bib-0003] Ahmadia, G. N. , Pezold, F. L. , & Smith, D. J. (2012). Cryptobenthic fish biodiversity and microhabitat use in healthy and degraded coral reefs in SE Sulawesi, Indonesia. Marine Biodiversity, 42, 433–442.

[ece33288-bib-0004] Airoldi, L. , Turon, X. , Perkol‐Finkel, S. , & Rius, M. (2015). Corridors for aliens but not for natives: Effects of marine urban sprawl at a regional scale. Diversity and Distributions, 21, 755–768.

[ece33288-bib-0005] Aronson, M. F. , La Sorte, F. A. , Nilon, C. H. , Katti, M. , Goddard, M. A. , Lepczyk, C. A. , … Dobbs, C. , et al. (2014). A global analysis of the impacts of urbanization on bird and plant diversity reveals key anthropogenic drivers. Proceedings of the Royal Society B, 281, 20133330. 24523278 10.1098/rspb.2013.3330 PMC4027400

[ece33288-bib-0006] Bates, D. , Maechler, M. , Bolker, B. , & Walker, S. (2015). Fitting linear mixed‐effects models using lme4. Journal of Statistical Software, 67, 1–48.

[ece33288-bib-0007] Blair, R. B. (1999). Birds and butterflies along an urban gradient: Surrogate taxa for assessing biodiversity? Ecological Applications, 9, 164–170.

[ece33288-bib-0008] Brandl, S. J. , & Bellwood, D. R. (2016). Microtopographic refuges shape consumer‐producer dynamics by mediating consumer functional diversity. Oecologia, 182, 203–217. 27147547 10.1007/s00442-016-3643-0

[ece33288-bib-0009] Bulleri, F. , & Chapman, M. G. (2010). The introduction of coastal infrastructure as a driver of change in marine environments. Journal of Applied Ecology, 47, 26–35.

[ece33288-bib-0010] Bulleri, F. , Chapman, M. G. , & Underwood, A. J. (2005). Intertidal assemblages on seawalls and vertical rocky shores in Sydney Harbour, Australia. Austral Ecology, 30, 655–667.

[ece33288-bib-0011] Burt, J. A. , Feary, D. A. , Cavalcante, G. , Bauman, A. G. , & Usseglio, P. (2013). Urban breakwaters as reef fish habitat in the Persian Gulf. Marine Pollution Bulletin, 72, 342–350. 23154139 10.1016/j.marpolbul.2012.10.019

[ece33288-bib-0012] Cenci, E. , Pizzolon, M. , Chimento, N. , & Mazzoldi, C. (2011). The influence of a new artificial structure on fish assemblages of adjacent hard substrata. Estuarine, Coastal and Shelf Science, 91, 133–149.

[ece33288-bib-0013] Chace, J. F. , & Walsh, J. J. (2006). Urban effects on native avifauna: A review. Landscape and Urban Planning, 74, 46–69.

[ece33288-bib-0014] Chao, A. , & Jost, L. (2012). Coverage‐based rarefaction and extrapolation: Standardizing samples by completeness rather than size. Ecology, 93, 2533–2547. 23431585 10.1890/11-1952.1

[ece33288-bib-0015] Clynick, B. G. , Chapman, M. G. , & Underwood, A. J. (2007). Effects of epibiota on assemblages of fish associated with urban structures. Marine Ecology Progress Series, 332, 201–210.

[ece33288-bib-0016] Clynick, B. G. , Chapman, M. G. , & Underwood, A. J. (2008). Fish assemblages associated with urban structures and natural reefs in Sydney, Australia. Austral Ecology, 33, 140–150.

[ece33288-bib-0017] Connell, S. D. (2001). Urban structures as marine habitats: An experimental comparison of the composition and abundance of subtidal epibiota among pilings, pontoons and rocky reefs. Marine Environmental Research, 52, 115–125. 11525426 10.1016/s0141-1136(00)00266-x

[ece33288-bib-0018] Dafforn, K. A. , Glasby, T. M. , Airoldi, L. , Rivero, N. K. , Mayer‐Pinto, M. , & Johnston, E. L. (2015). Marine urbanization: An ecological framework for designing multifunctional artificial structures. Frontiers in Ecology and the Environment, 13, 82–90.

[ece33288-bib-0019] D'Croz, L. , Del Rosario, J. B. , & Gondola, P. (2005). The effect of fresh water runoff on the distribution of dissolved inorganic nutrients and plankton in the Bocas del Toro Archipelago, Caribbean Panama. Caribbean Journal of Science, 41, 414–429.

[ece33288-bib-0020] Dearborn, D. C. , & Kark, S. (2010). Motivations for conserving urban biodiversity. Conservation Biology, 24, 432–440. 19775276 10.1111/j.1523-1739.2009.01328.x

[ece33288-bib-0021] Depczynski, M. , & Bellwood, D. R. (2003). The role of cryptobenthic reef fishes in coral reef trophodynamics. Marine Ecology Progress Series, 256, 183–191.

[ece33288-bib-0022] Dornelas, M. , Gotelli, N. J. , McGill, B. , Shimadzu, H. , Moyes, F. , Sievers, C. , & Magurran, A. E. (2014). Assemblage time series reveal biodiversity change but not systematic loss. Science, 344, 296–299. 24744374 10.1126/science.1248484

[ece33288-bib-0023] Easson, C. G. , Matterson, K. O. , Freeman, C. J. , Archer, S. K. , & Thacker, R. W. (2015). Variation in species diversity and functional traits of sponge communities near human populations in Bocas del Toro, Panama. PeerJ, 3, 1385. 10.7717/peerj.1385 PMC4647605 26587347

[ece33288-bib-0024] Ferrario, F. , Iveša, L. , Jaklin, A. , Perkol‐Finkel, S. , & Airoldi, L. (2016). The overlooked role of biotic factors in controlling the ecological performance of artificial marine habitats. Journal of Applied Ecology, 53, 16–24.

[ece33288-bib-0025] Fowler, A. M. , & Booth, D. J. (2013). Seasonal dynamics of fish assemblages on breakwaters and natural rocky reefs in a temperate estuary: Consistent assemblage differences driven by sub‐adults. PLoS ONE, 8, e75790. 24086634 10.1371/journal.pone.0075790 PMC3784400

[ece33288-bib-0026] Freestone, A. L. , & Inouye, B. D. (2015). Nonrandom community assembly and high temporal turnover promote regional coexistence in tropics but not temperate zone. Ecology, 96, 264–273. 26236911 10.1890/14-0145.1

[ece33288-bib-0027] Freestone, A. L. , & Osman, R. W. (2011). Latitudinal variation in local interactions and regional enrichment shape patterns of marine community diversity. Ecology, 92, 208–217. 21560691 10.1890/09-1841.1

[ece33288-bib-0028] Freestone, A. L. , Osman, R. W. , Ruiz, G. M. , & Torchin, M. E. (2011). Stronger predation in the tropics shapes species richness patterns in marine communities. Ecology, 92, 983–993. 21661559 10.1890/09-2379.1

[ece33288-bib-0029] Freestone, A. L. , Ruiz, G. M. , & Torchin, M. E. (2013). Stronger biotic resistance in tropics relative to temperate zone: Effects of predation on marine invasion dynamics. Ecology, 94, 1370–1377. 23923500 10.1890/12-1382.1

[ece33288-bib-0030] Gaston, K. J. (2000). Global patterns in biodiversity. Nature, 405, 220–227. 10821282 10.1038/35012228

[ece33288-bib-0031] Gaston, K. J. , Ávila‐Jiménez, M. L. , & Edmondson, J. L. (2013). Managing urban ecosystems for goods and services. Journal of Applied Ecology, 50, 830–840.

[ece33288-bib-0032] Glasby, T. , & Connell, S. (2001). Orientation and position of substrata have large effects on epibiotic assemblages. Marine Ecology Progress Series, 214, 127–135.

[ece33288-bib-0033] Goatley, C. H. R. , & Brandl, S. J. (2017). Cryptobenthic reef fishes. Current Biology, 27, R452–R454. https://doi.org/10.1016/j.cub.2017.03.051 28586677 10.1016/j.cub.2017.03.051

[ece33288-bib-0034] Goatley, C. H. R. , González‐Cabello, A. , & Bellwood, D. R. (2016). Reef‐scale partitioning of cryptobenthic fish assemblages across the Great Barrier Reef, Australia. Marine Ecology Progress Series, 544, 271–280.

[ece33288-bib-0035] González‐Cabello, A. , & Bellwood, D. R. (2009). Local ecological impacts of regional biodiversity on reef fish assemblages. Journal of Biogeography, 36, 1129–1137.

[ece33288-bib-0036] Grimm, N. B. , Faeth, S. H. , Golubiewski, N. E. , Redman, C. L. , Wu, J. , Bai, X. , & Briggs, J. M. (2008). Global change and the ecology of cities. Science, 319, 756–760. 18258902 10.1126/science.1150195

[ece33288-bib-0037] Hillebrand, H. (2004). On the generality of the latitudinal diversity gradient. The American Naturalist, 163, 192–211. 10.1086/381004 14970922

[ece33288-bib-0138] Hundt, P.J. , Hundt, M.J. , Staley, C. , Sadowsky, M.J. , and Simons, A.M. (2017). The Diet and Gut Microbial Communities of Two Closely Related Combtooth Blennies, Chasmodes saburrae and Scartella cristata. Copeia. 105(2), 249–25.

[ece33288-bib-0038] Hsieh, T. , Ma, K. , & Chao, A. (2016). iNEXT: An R package for rarefaction and extrapolation of species diversity (Hill numbers). Methods in Ecology and Evolution. 7, 1451–1456. https://doi.org/10.1111/2041-210X.12613

[ece33288-bib-0039] Jud, Z. R. , Layman, C. A. , Lee, J. A. , & Arrington, D. A. (2011). Recent invasion of a Florida (USA) estuarine system by lionfish Pterois volitans/P. miles. Aquatic Biology, 13, 21–26.

[ece33288-bib-0040] Kennish, M.J. , & Paerl, H.W. (2010). Coastal lagoons: Critical habitats of environmental change, Boca Raton, FL: CRC Press.

[ece33288-bib-0041] Leray, M. , & Knowlton, N. (2015). DNA barcoding and metabarcoding of standardized samples reveal patterns of marine benthic diversity. Proceedings of the National Academy of Sciences, 112, 2076–2081. 10.1073/pnas.1424997112 PMC4343139 25646458

[ece33288-bib-0042] Lockett, M. M. , & Gomon, M. F. (2001). Ship mediated fish invasions in Australia: Two new introductions and a consideration of two previous invasions. Biological Invasions, 3, 187–192.

[ece33288-bib-0043] McDonnell, M. J. , & Pickett, S. T. (1990). Ecosystem structure and function along urban‐rural gradients: An unexploited opportunity for ecology. Ecology, 71, 1232–1237.

[ece33288-bib-0044] McKinney, M. L. (2008). Effects of urbanization on species richness: A review of plants and animals. Urban Ecosystems, 11, 161–176.

[ece33288-bib-0045] Moreau, S. , Péron, C. , Pitt, K. A. , Connolly, R. M. , Lee, S. Y. , & Meziane, T. (2008). Opportunistic predation by small fishes on epibiota of jetty pilings in urban waterways. Journal of Fish Biology, 72, 205–217.

[ece33288-bib-0046] Niemelä, J. (1999). Ecology and urban planning. Biodiversity & Conservation, 8, 119–131.

[ece33288-bib-0047] Oksanen, J. , Kindt, R. , Legendre, P. , O'Hara, B. , Stevens, M.H.H. , Oksanen, M.J. , & Suggests, M. (2007). The vegan package. Community Ecology Package, 10, pp. 631–637.

[ece33288-bib-0048] Osman, R. W. (2015). Regional variation in the colonization of experimental substrates by sessile marine invertebrates: Local vs. regional control of diversity. Journal of Experimental Marine Biology and Ecology, 473, 227–286.

[ece33288-bib-0049] Pianka, E.R. (1966). Latitudinal gradients in species diversity: A review of concepts. American Naturalist, 3, 3–46.

[ece33288-bib-0050] Ricklefs, R.E. , & Schluter, D. (1993). Species Diversity in Ecological Communities: Historical and Geographical Perspectives, Chicago, IL: University of Chicago Press.

[ece33288-bib-0051] Rilov, G. , & Benayahu, Y. (1998). Vertical artificial structures as an alternative habitat for coral reef fishes in disturbed environments. Marine Environmental Research, 45, 431–451.

[ece33288-bib-0052] Robertson, D.R. , & Van Tassell, J. (2015). Shorefishes of the Greater Caribbean: Online information system. Version 1.0, Balboa, Panamá: Smithsonian Tropical Research Institute.

[ece33288-bib-0053] Rodemann, J.R. , & Brandl, S.J. (2017). Consumption pressure in coastal marine environments decreases with latitude and in artificial vs. natural habitats. Marine Ecology Progress Series, 574, 167–179. https://doi.org/10.3354/meps12170

[ece33288-bib-0054] Rogers, T. L. , Byrnes, J. E. , & Stachowicz, J. J. (2016). Native predators limit invasion of benthic invertebrate communities in Bodega Harbour, California, USA. Marine Ecology Progress Series, 545, 161–173.

[ece33288-bib-0055] Roy, K. , Jablonski, D. , Valentine, J. W. , & Rosenberg, G. (1998). Marine latitudinal diversity gradients: Tests of causal hypotheses. Proceedings of the National Academy of Sciences USA, 95, 3699–3702. 10.1073/pnas.95.7.3699 PMC19899 9520429

[ece33288-bib-0156] Rützler, K. , Macintyre, IG. , (1982) *The Atlantic Barrier Reef Ecosystem at Carrie Bow Cay, Belize: I. Structure and Communities* Smithsonian Institution Press: Washington, DC, p 539

[ece33288-bib-0056] Ruiz, G.M. , Freestone, A.L. , Fofonoff, P.W. , & Simkanin, C. (2009) *Habitat distribution and heterogeneity in marine invasion dynamics: The importance of hard substrate and artificial structure* In Wahl M. (Ed.), Marine hard bottom communities (pp. 321–332). Springer‐Verlag: Berlin.

[ece33288-bib-0057] Shochat, E. , Lerman, S. B. , Anderies, J. M. , Warren, P. S. , Faeth, S. H. , & Nilon, C. H. (2010). Invasion, Competition, and Biodiversity Loss in Urban Ecosystems. BioScience, 60, 199–208.

[ece33288-bib-0058] Simkanin, C. , Dower, J. F. , Filip, N. , Jamieson, G. , & Therriault, T. W. (2013). Biotic resistance to the infiltration of natural benthic habitats: Examining the role of predation in the distribution of the invasive ascidian *Botrylloides violaceus* . Journal of Experimental Marine Biology and Ecology, 439, 76–83.

[ece33288-bib-0059] Srivastava, D. S. (1999). Using local–regional richness plots to test for species saturation: Pitfalls and potentials. Journal of Animal Ecology, 68, 1–16.

[ece33288-bib-0060] Van Tassell, J.L. , Aiken, K.A. , & Tornabene, L. (2015) Gobiosoma spilotum. The IUCN Red List of Threatened Species 2015: e.T186016A1804288.

[ece33288-bib-0061] Wonham, M. J. , Carlton, J. T. , Ruiz, G. M. , & Smith, L. D. (2000). Fish and ships: Relating dispersal frequency to success in biological invasions. Marine Biology, 136, 1111–1121.

