# Supplementary material for: Marine dock pilings foster diverse, native cryptobenthic fish assemblages across bioregions
Source: Ecol Evol. 2017 Jul 31;7(17):7069–79. doi: 10.1002/ece3.3288 (PMC5587495; doi:10.1002/ece3.3288)
Supplement: Supplementary file 1 [file ECE3-7-7069-s001.pdf]

Supplemental Information

Title: Marine dock pilings foster diverse, native cryptobenthic fish assemblages across bioregions

Authors: Simon J. Brandl[1]*, Jordan M. Casey[2], Nancy Knowlton[2], J. Emmett Duffy[1]

[1]Tennenbaum Marine Observatories Network, Smithsonian Institution, Edgewater, Maryland 21037, USA

[2]Department of Invertebrate Zoology, National Museum of Natural History, Smithsonian Institution, Washington, DC 20013

Email addresses: brandls@si.edu, caseyj@si.edu, knowlton@si.edu, duffye@si.edu

Table S1: Species collected from dock pilings at each of the six locations. Bold species names indicate species identified by the SIMPER as the top three species contributing to dissimilarities in pairwise comparisons among locations.

Panama	Belize	Florida	North Carolina	Massachusetts	Maine
A. aspera	*A. saxatilis*	*A. virginicus*	*D. holbrooki*	***M. aeneus***	*C. lumpus*
C. rostrata	*Blenniidae sp1*	*A. probatoceph.*	*G. bosc*	***P. gunnellus***	***M. aeneus***
C. cruentata	*C. rostrata*	*B. soporator*	***H. geminatus***	*T. onitis*	***P. gunnellus***
C. capistratus	*E. nigricans*	*C. boleosoma*	*H. hentz*	***T. adspersus***	***T. adspersus***
C. glaucofraenum	***H. pseudoaequip.***	*D. holbrooki*	*H. ionthas*		
G. spilotum	*H. springeri*	*G. bosc*	*H. sp1*		
H. bivittatus	*Labridae sp1*	*H. bermudensis*	***L. rhomboides***		
H. pseudoaequip.	*L. bucciferus*	***H. geminatus***	*O. tau*		
L. crocodilus	*M. delalandii*	***H. pseudoaequip.***	*O. chrysoptera*		
P. marmoreus	*M. macropus*	*L. kalisherae*	*P. marmoreus*		
P. nigripinnis	*M. bonaci*	*L. nicholsi*			
R. ruber	*P. marmoreus*	*M. bonaci*			
S. occidentalis	*P. nigripinnis*	*P. marmoreus*			
S. adustus	*S. iserti*	*P. volitans*			
	S. viride	***S. cristata***			
	S. nanodes	*Scaridae sp1*			
	S. occidentalis	*Scorpaenidae sp1*			
	Starksia sp1	*S. ocellata*			
	S. leucostictus	*T. macrodon*			
	S. xanthurus				
	T. bifasciatum				

Table S2: Most important species determining distances among assemblages as provided by the SIMPER analysis. Only the three most influential species are listed for each pairwise comparison. Bel. = Belize; Pan. = Panama.

Comparison	*Species 1*	*%*	*Species 2*	*%*	*Species 3*	*%*
Bel. docks vs. Bel. outcrops	*Coryphopterus personatus*	8.9	*Hypleurochilus pseudoaequipinnis*	8.4	*Acanthemblemaria aspera*	7.6
Bel. docks vs. Pan. docks	*Hypleurochilus pseudoaequipinnis*	25.9	*Stegastes adustus*	14.0	*Starksia occidentalis*	12.3
Bel. docks vs. Pan. outcrops	*Coryphopterus personatus*	56.6	*Hypleurochilus pseudoaequipinnis*	7.2	*Phaeoptyx pigmentaria*	3.1
Bel. outcrops vs. Pan. docks	*Coryphopterus personatus*	8.6	*Acanthemblemaria aspera*	7.2	*Hypleurochilus pseudoaequipinnis*	6.2
Bel. outcrops vs. Pan. docks	*Coryphopterus personatus*	43.9	*Aacanthemblemaria aspera*	4.3	*Enneanectyes atrorus*	2.6
Pan. docks vs. Pan. outcrops	*Coryphopterus personatus*	55.6	*Hypleurochilus pseudoaequipinnis*	5.2	*Stegastes adustus*	4.52

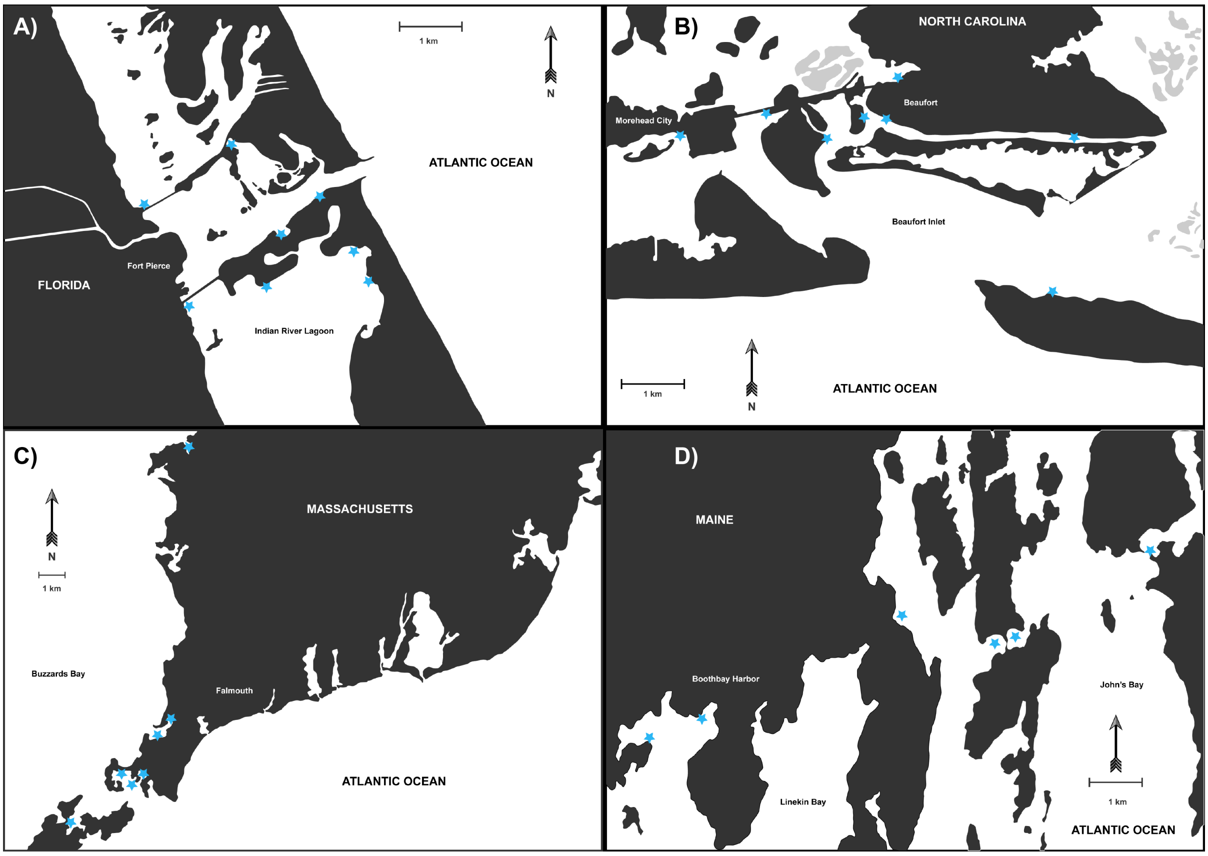

Figure S1: Location of sampled docks in Florida (A), North Carolina (B), Massachusetts (C), and Maine (D). Blue stars correspond to docks. Nearest population centres are indicated in white letters.

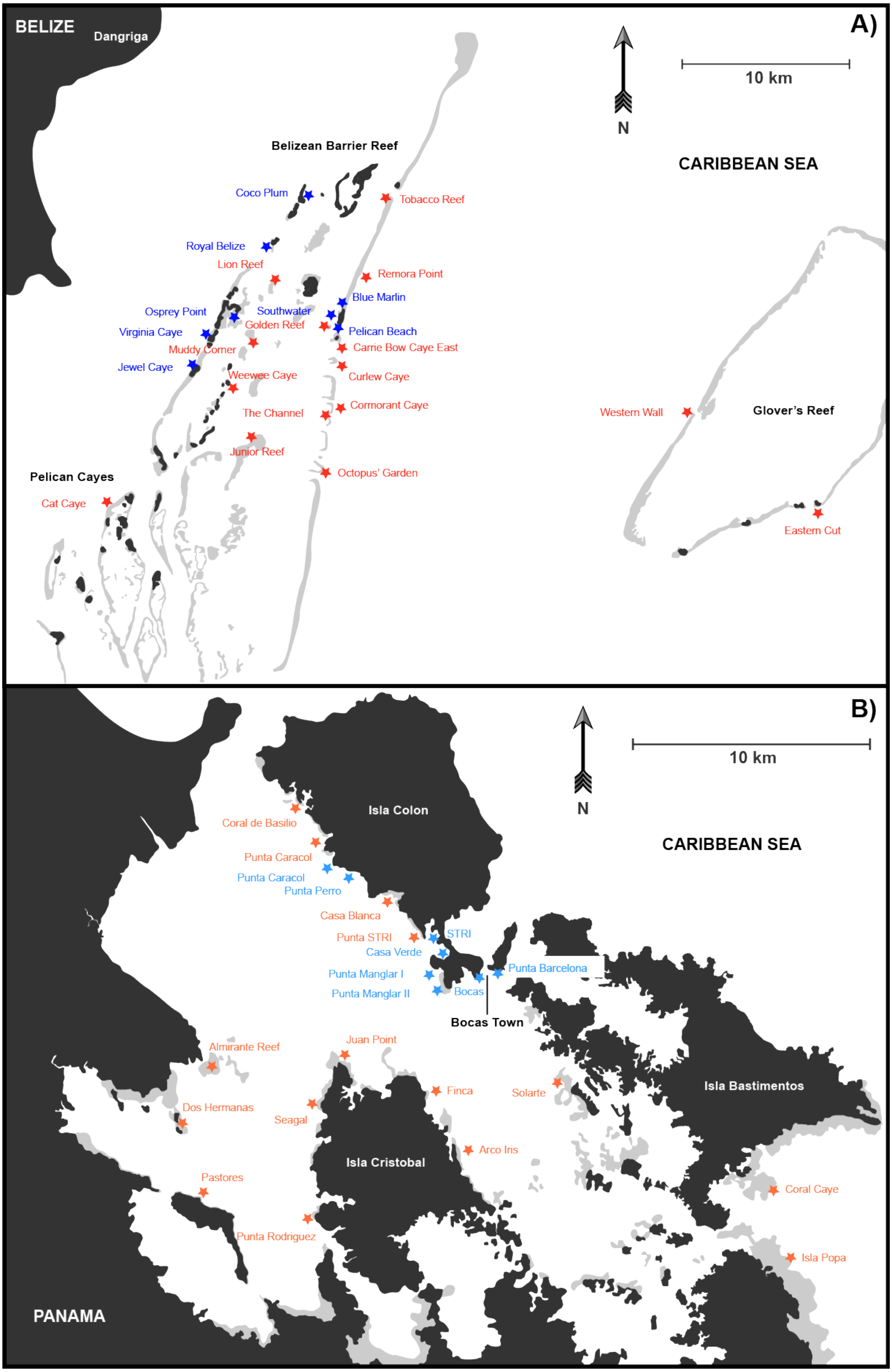
A)
BELIZE
Dangriga
10 km
N
CARIBBEAN SEA
Belizean Barrier Reef
Coco Plum
Tobacco Reef
Royal Belize
Lion Reef
Remora Point
Blue Marlin
Osprey Point
Southwater
Golden Reef
Virginia Caye
Pelican Beach
Carrie Bow Caye East
Muddy Corner
Jewel Caye
Curlew Caye
Weewee Caye
Cormorant Caye
The Channel
Western Wall
Glover's Reef
Junior Reef
Octopus' Garden
Pelican Cayes
Cat Caye
Eastern Cut
B)
10 km
N
CARIBBEAN SEA
Isla Colon
Coral de Basilio
Punta Caracol
Punta Caracol
Punta Perro
Casa Blanca
Punta STRI
STRI
Casa Verde
Punta Barcelona
Punta Manglar I
Punta Manglar II
Bocas
Bocas Town
Almirante Reef
Juan Point
Finca
Solarte
Dos Hermanas
Seagal
Isla Bastimentos
Arco Iris
Isla Cristobal
Pastores
Coral Caye
Punta Rodriguez
Isla Popa
PANAMA

Figure S2: Location of sampled docks and reef outcrops in Belize (A) and Panama (B). Dark and light blue stars correspond to docks. Red and orange stars correspond to reef outcrops. Nearest population centres (Dangriga in Belize, Bocas Town in Panama) are indicated.

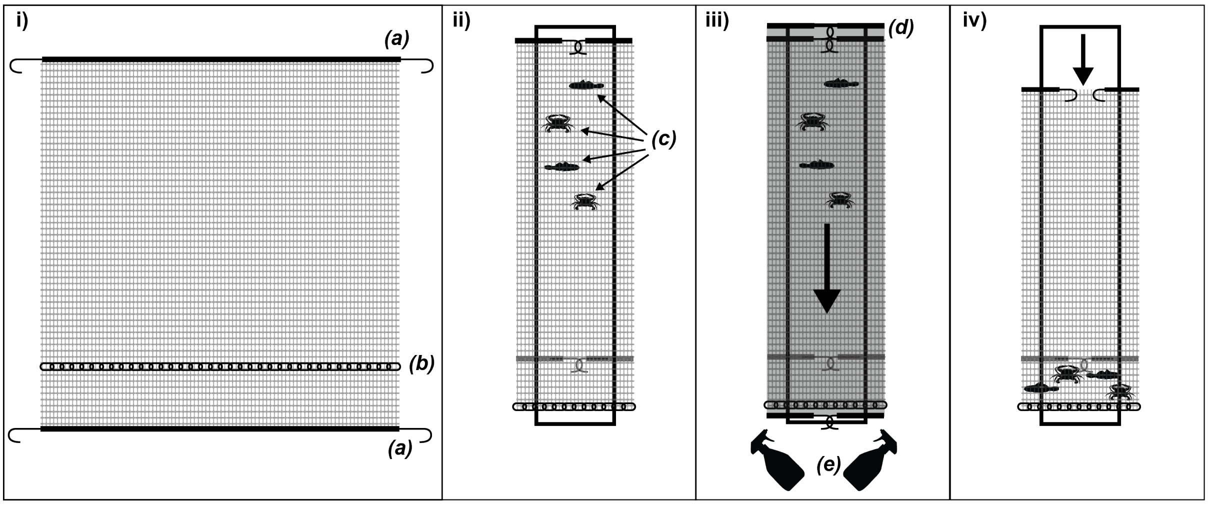

Figure S3: Description of the method applied to sample mobile cryptofauna from dock pilings. i) A small-mesh net is fitted with two bungee cords (a) on the top and bottom. Heavy chain is fixed to the net approximately 30 cm above the bottom bungee cord (b). ii) The net is wrapped around a piling with the bungee cords pulled tight, enclosing all mobile animals living on the piling within the net (c). A trough is formed through the weight of the chain. iii) An impermeable nylon tarp (d) is wrapped around the net and likewise tightened using bungee cords. Then, one liter of clove-oil:ethanol solution (1:5) is sprayed into the interior of the tarp (e), causing all anaesthetized animals to detach from the piling and sink to the bottom. iv) After removal of the tarp, the net can be rolled up from the top. Upon reaching the bottom bungee cord, the ends of the net can be folded in to prevent the loss of any animals in the trough.

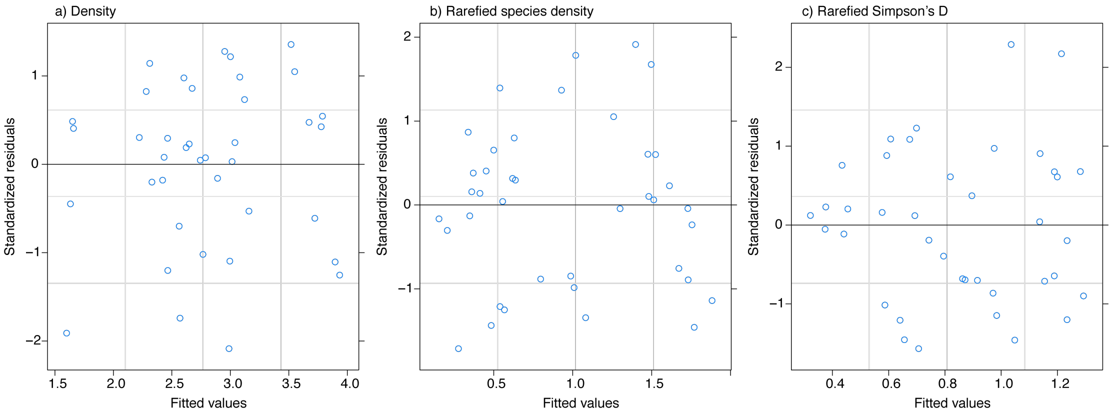

Fig S4: Residuals vs. fitted values for the three linear mixed models comparing density, species density and Simpson's D across latitude.

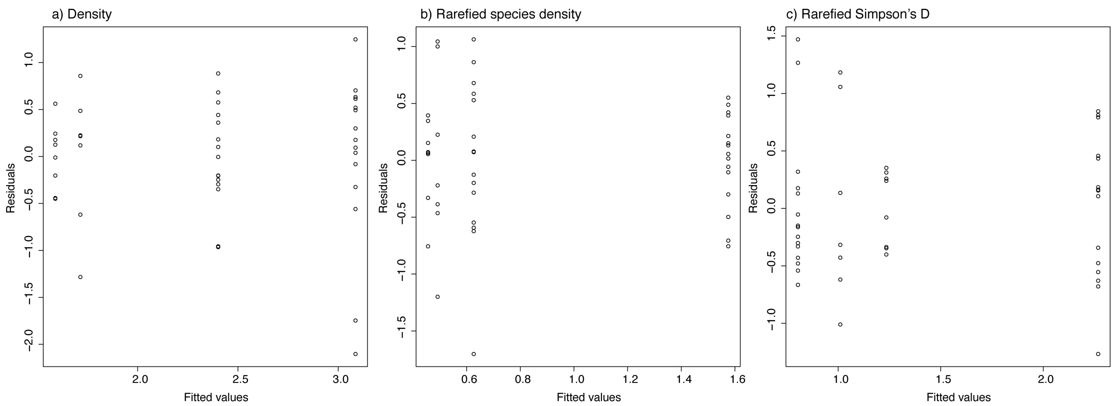

Fig S5: Residuals vs. fitted values for the three linear mixed models comparing density, species density and Simpson's D across locations and habitats.

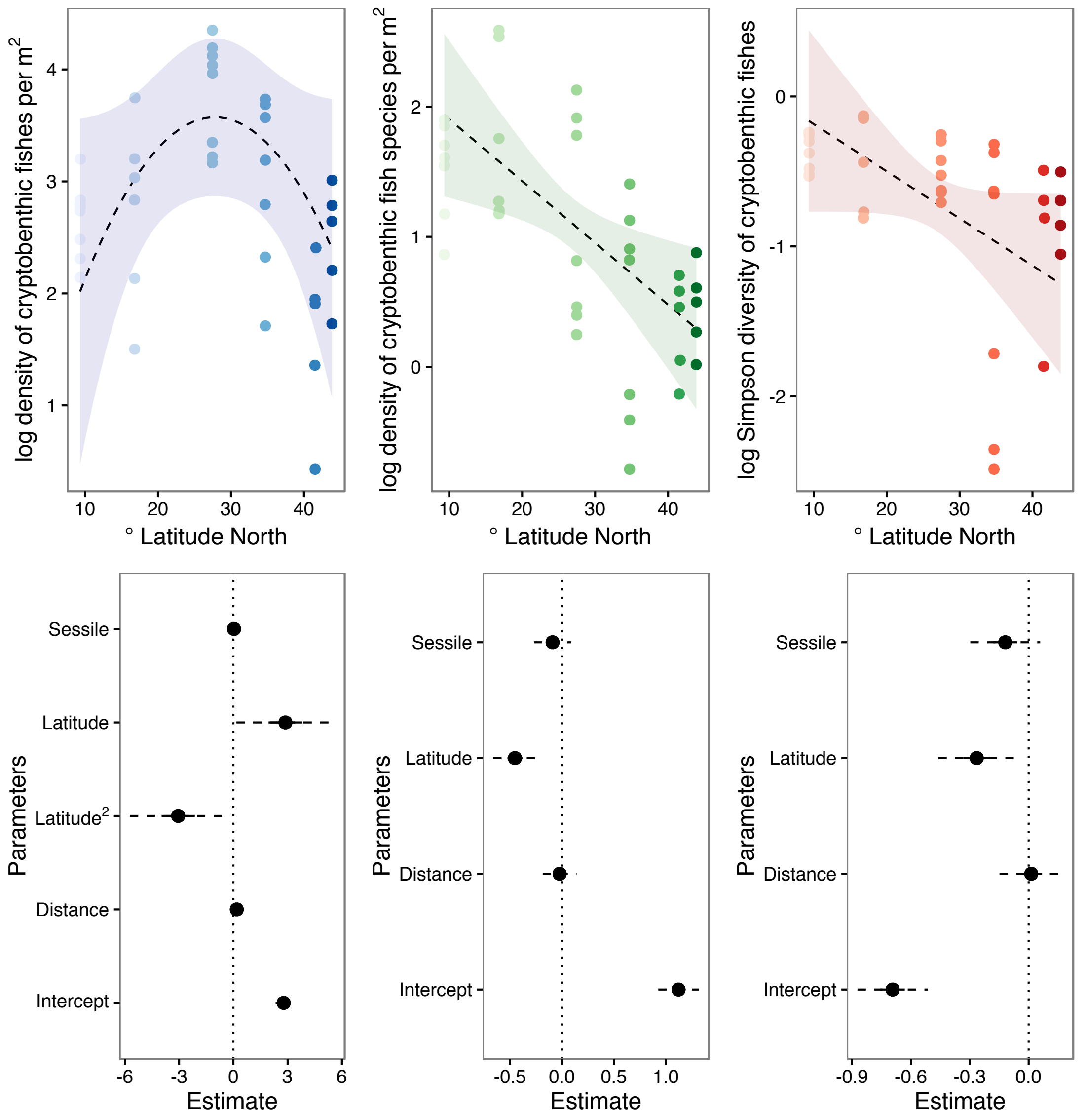

Fig S6: Model fits and coefficients for models run on raw data instead of rarefied species richness and Simpson's D estimates, respectively.

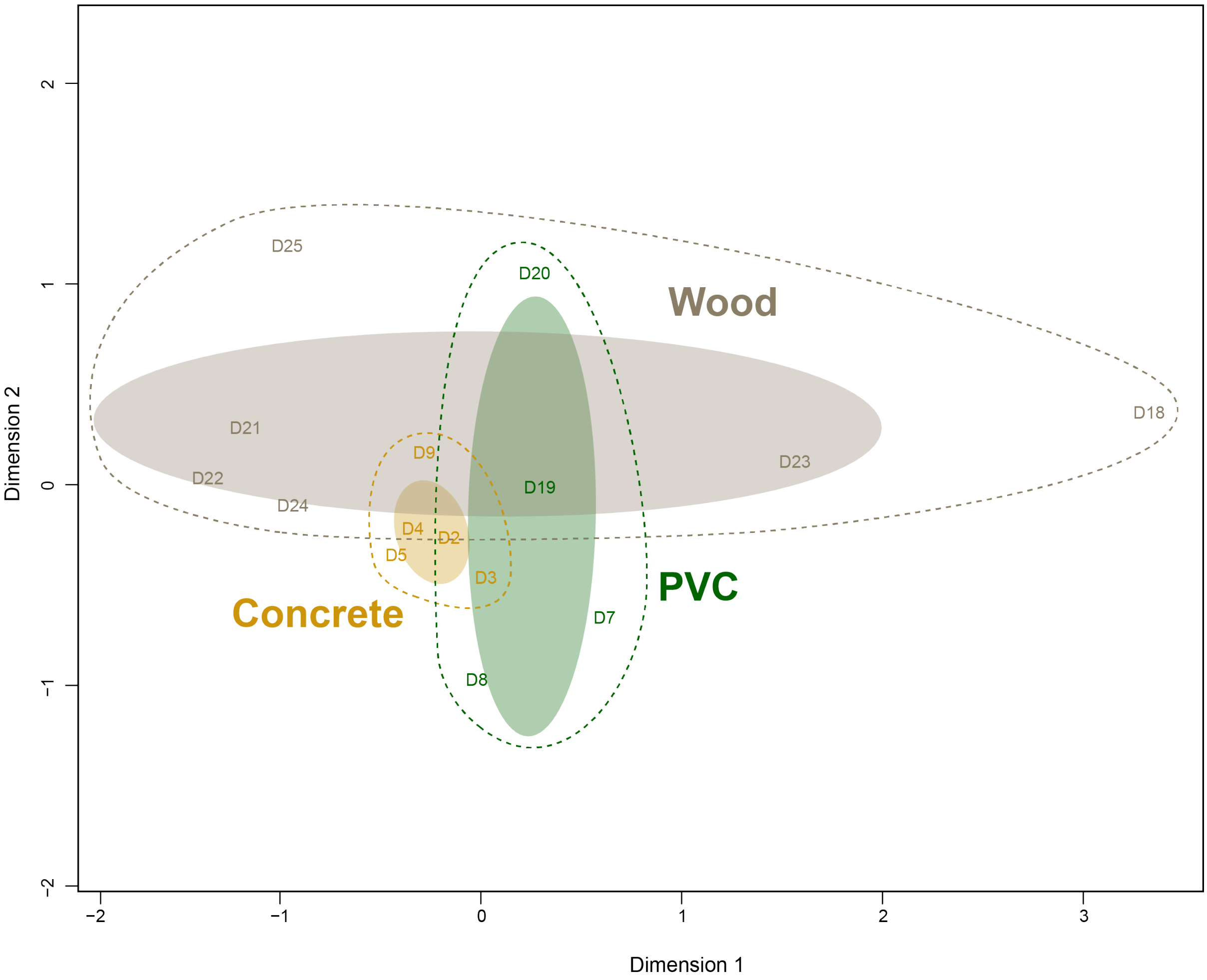

Fig. S7: nMDS ordination showing separation between communities from dock pilings built from different materials. The dark tan ellipse represents wooden pilings (not found in Panama), the light tan ellipse represents concrete pilings (not found in Belize), while the green ellipse represents PVC pilings present in both locations.
